# Enzymatic diversity of the *Clostridium thermocellum* cellulosome is crucial for the degradation of crystalline cellulose and plant biomass

**DOI:** 10.1038/srep35709

**Published:** 2016-10-19

**Authors:** Katsuaki Hirano, Masahiro Kurosaki, Satoshi Nihei, Hiroki Hasegawa, Suguru Shinoda, Mitsuru Haruki, Nobutaka Hirano

**Affiliations:** 1 Department of Chemical Biology & Applied Chemistry, College of Engineering, Nihon University, Koriyama, Fukushima 963-8642, Japan

## Abstract

The cellulosome is a supramolecular multienzyme complex comprised of a wide variety of polysaccharide-degrading enzymes and scaffold proteins. The cellulosomal enzymes that bind to the scaffold proteins synergistically degrade crystalline cellulose. Here, we report *in vitro* reconstitution of the *Clostridium thermocellum* cellulosome from 40 cellulosomal components and the full-length scaffoldin protein that binds to nine enzyme molecules. These components were each synthesized using a wheat germ cell-free protein synthesis system and purified. Cellulosome complexes were reconstituted from 3, 12, 30, and 40 components based on their contents in the native cellulosome. The activity of the enzyme-saturated complex indicated that greater enzymatic variety generated more synergy for the degradation of crystalline cellulose and delignified rice straw. Surprisingly, a less complete enzyme complex displaying fewer than nine enzyme molecules was more efficient for the degradation of delignified rice straw than the enzyme-saturated complex, despite the fact that the enzyme-saturated complex exhibited maximum synergy for the degradation of crystalline cellulose. These results suggest that greater enzymatic diversity of the cellulosome is crucial for the degradation of crystalline cellulose and plant biomass, and that efficient degradation of different substrates by the cellulosome requires not only a different enzymatic composition, but also different cellulosome structures.

The cellulosome is a supramolecular multienzyme complex comprised of a wide variety of polysaccharide-degrading enzymes (e.g., cellulases, hemicellulases, and pectinases) and scaffold proteins, and is displayed on the cell surface of anaerobic cellulolytic bacteria^1^,^2^. *Clostridium thermocellum* is one of the most investigated cellulosome-producing anaerobic bacteria. Cellulosome formation by *C. thermocellum* is mediated by two specific interactions; one interaction is between the type-I dockerin module at the C-terminus of cellulosomal components and the internal nine type-I cohesin modules of the primary scaffoldin protein, CipA, and the other is mediated between the type-II dockerin module at the C-terminus of CipA and the internal type-II cohesin modules of the cell-surface-displayed and unbound secondary scaffoldin proteins. The efficient degradation of crystalline cellulose by *C. thermocellum* is essentially dependent on the formation of a supramolecular cellulosome complex mediated by CipA, which contains a carbohydrate-binding module (CBM) belonging to family 3a (CBM3a) that interacts with crystalline cellulose^3^,^4^, whereas the lack of the secondary scaffoldin proteins has almost no effect on the efficient degradation of crystalline cellulose by *C. thermocellum*^5^.

The genome of *C. thermocellum* ATCC 27405 contains at least 79 cellulosomal genes, of which ~70 encode the type-I dockerin-containing proteins. *C. thermocellum* cellulosomes isolated from cells grown on different carbon sources have different enzymatic compositions, as revealed by proteomic studies^6^,^7^. The enzymatic composition of the *C. thermocellum* cellulosome isolated from cells grown on crystalline cellulose has been reported in terms of relative ratios of normalized spectral abundance factors (NSAF) of each cellulosomal component^7^; the NSAF values of proteins have been used for determining the relative protein ratios in a multiprotein complex^8^. Based on the reported ratios of the NSAF values of the cellulosomal components, we recently reconstituted the cellulosome complex using full-length CipA and the three major cellulosomal cellulases, Cel48S, Cel8A, and Cel9K, at a molar ratio of Cel48S:Cel8A:Cel9K = 4.06:1.82:0.72^9^. However, the cellulosome complex comprised of these three major cellulases was estimated to exhibit significantly lower activity for crystalline cellulose than the native cellulosome, suggesting that greater enzymatic variety in the cellulosome complex may be essential for the high activity for crystalline cellulose exhibited by the native cellulosome. In fact, the activity of the cellulosome complex reconstituted from full-length CipA and the protein mixture secreted from *cipA*-deficient *C. thermocellum* cells showed ~80% of the activity for crystalline cellulose of the native cellulosome^10^. Thus, to elucidate the mechanism responsible for the high activity for crystalline cellulose of the *C. thermocellum* cellulosome, it is necessary to perform *in vitro* reconstitution of the supramolecular cellulosome from a wide variety of the purified cellulosomal components. However, the reconstitution of supramolecular cellulosomes with greater enzymatic variety requires the preparation of large scaffoldin proteins and dozens of cellulosomal components.

A wheat germ cell-free protein synthesis system using purified wheat embryos is suitable for synthesizing a large set of artificial multidomain proteins^11^ and for producing large cellulosomal proteins that are difficult to produce using recombinant *Escherichia coli*^9^,^12^. High-throughput synthesis of the 42 components (excluding CipA) of the *C. thermocellum* cellulosome and a large set of fusion proteins of *C. thermocellum* Cel5E and various CBMs using the wheat germ system have also been reported^13^,^14^. Furthermore, we recently succeeded in reconstituting the *C. thermocellum* cellulosome complex from the full-length scaffoldin protein and three cellulosomal cellulases using the wheat germ system^9^; this study demonstrated stoichiometric assembly of the cellulosome complex reconstituted from full-length CipA. Therefore, the wheat germ system makes it possible to reconstitute a supramolecular cellulosome complex from a full-length scaffoldin protein and dozens of cellulosomal components. Herein, we report the *in vitro* reconstitution of the *C. thermocellum* cellulosome from 40 cellulosomal components and the full-length scaffoldin protein, which were synthesized using the wheat germ cell-free system and purified; we also report the effect of the enzymatic diversity in the cellulosome complex on its activity for crystalline cellulose and plant biomass. This is the first report on the *in vitro* reconstitution of a supramolecular cellulosome complex comprised of a full-length scaffoldin protein and dozens of purified components.

## Results

### Cell-Free Protein Synthesis and Purification of Cellulosomal Components

**Figure 1:**
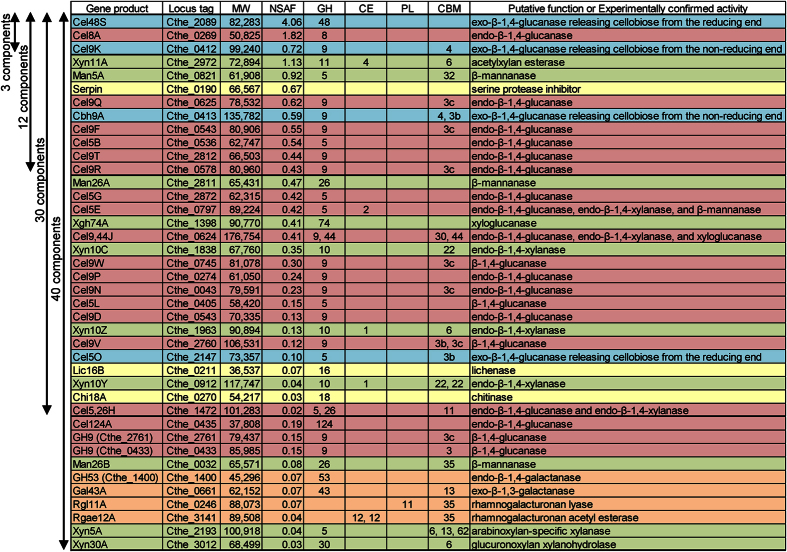
Cellulosomal components used for the *in vitro* reconstitution of the cellulosome. The 40 cellulosomal components used for the reconstitution of the cellulosome complexes are arranged in approximately descending order of the reported ratios of normalized spectral abundance factors (NSAF) of each cellulosomal component in the native cellulosome isolated from cells grown on crystalline cellulose^7^; although the figure includes some exceptions, such as the last 10 components, the enzymatic activities and the gene product names of most of these were recently assigned in the carbohydrate-active enzymes (CAZy) database. The 3, 12, 30, and 40 cellulosomal components used for the reconstitution of the cellulosome complexes are indicated. GH, CE, PL, and CBM denote glycoside hydrolase family, carbohydrate esterase family, polysaccharide lyase family, and carbohydrate-binding module family, respectively. Exoglucanases, endoglucanases, hemicellulases, pectic enzymes, and other proteins are shown in blue, red, green, brown, and yellow, respectively.

To reconstitute the *C. thermocellum* cellulosome, we selected 40 type-I dockerin-containing proteins that were previously identified by proteomic analysis as components of the native cellulosome isolated from cells grown on crystalline cellulose^7^ (Fig. 1): 23 cellulases, which include 4 exoglucanases (Cel48S^15^, Cel9K^16^, Cbh9A^17^, and Cel5O^18^) and 19 endoglucanases (Cel8A^19^, Cel9Q^20^, Cel9F^21^, Cel5B^22^, Cel9T^23^, Cel9R^24^, Cel5G^25^, Cel5E^26^, Cel9,44J^27^, Cel9W, Cel9P, Cel9N^28^, Cel5L, Cel9D^29^, Cel9V, Cel5,26H^30^, Cel124A^31^, GH9 (Cthe_2761), and GH9 (Cthe_0433)); 10 hemicellulases, which include six xylanases (Xyn11A^32^, Xyn10C^33^, Xyn10Z^34^, Xyn10Y^35^, Xyn5A^36^, and Xyn30A^37^), three mannanases (Man5A^38^, Man26A^39^, and Man26B^40^), and one xyloglucanase (Xgh74A^41^); four pectic enzymes, which include two galactanases (GH53 (Cthe_1400) and Gal43A^42^), one rhamnogalacturonan lyase (Rgl11A^43^), and one rhamnogalacturonan acetyl esterase (Rgae12A); and three other proteins, which include one lichenase (Lic16B^44^), one chitinase (Chi18A^45^), and one protease inhibitor (serpin^46^). Proteins with unknown functions previously identified as cellulosomal components were not used in this study. In Fig. 1, these 40 cellulosomal components are arranged in approximately descending order of the reported NSAF values of each cellulosomal component^7^; although there are some exceptions, such as the last 10 components, the enzymatic activities and the gene product names of most of these components were recently assigned in the carbohydrate-active enzymes (CAZy) database. We synthesized the 40 cellulosomal proteins as glutathione S-transferase (GST) fusion proteins using a wheat germ cell-free protein synthesis system, and the cell-free synthesized GST fusion proteins were purified by glutathione affinity chromatography. The purified proteins were confirmed by SDS-polyacrylamide gel electrophoresis (SDS-PAGE) (Supplementary Fig. S1) and western blotting analysis using an antibody against the C-terminal FLAG tag of the cellulosomal components (Supplementary Fig. S2). The amount of cellulosomal components purified from 1 ml of translation mixture was between 1 and 10 μg. The binding of each cellulosomal component (excluding Cel124A) to scaffoldin protein was confirmed by an electrophoretic mobility shift assay of miniscaffoldin protein, ΔCipA, which contains two type-I cohesin modules and a CBM3a, using an antibody against the C-terminal Strep tag of ΔCipA (Supplementary Fig. S3); low-affinity binding of the type-I dockerin module of Cel124A to the type-I cohesin module of CipA was reported previously^47^. The activity reported as the major enzymatic activity of each cellulosomal enzyme (excluding Gal43A) or the activity expected from the protein domain of each cellulosomal enzyme were confirmed (Supplementary Fig. S4); Gal43A (exo-*β*-1,3-galactanase) acts only on a *β*-1,3-linked galactose chain and does not hydrolyze *β*-1,4-linked galactose-containing polysaccharides^42^, such as potato galactan.

### Activity of *In Vitro* Reconstituted Cellulosome

**Figure 2:**
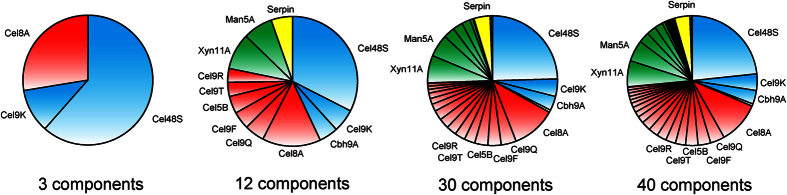
Protein contents in each enzyme mixture used for the *in vitro* reconstitution of cellulosome complexes. The cellulosomal components used for the reconstitution of cellulosome complexes were mixed at a molar ratio based on the reported ratios of the NSAF values of each cellulosomal component^7^, shown in Fig. 1. The molar ratios of each cellulosomal protein in the 3, 12, 30, and 40 enzyme mixes used for the reconstitution of the cellulosome complexes are shown, with the top 12 proteins in Fig. 1 indicated. Exoglucanases, endoglucanases, hemicellulases, pectic enzymes, and other proteins are shown in blue, red, green, brown, and yellow, respectively.

To investigate the effect of enzymatic variety in the cellulosome complex on its activity, we stoichiometrically assembled the complexes by mixing 3, 12, 30, or 40 cellulosomal proteins and full-length scaffoldin protein at a molar ratio of CipA/enzyme = 1/9 (cohesin/dockerin = 1/1); stoichiometric assembly generates maximum synergy for the degradation of crystalline cellulose^9^. Here, “CipA/enzyme” denotes the molar ratio of full-length CipA containing nine cohesin modules relative to the cellulosomal component containing one dockerin module, and “cohesin/dockerin” denotes the molar ratio of the cohesin module relative to the dockerin module; for example, the complex assembled at CipA/enzyme = 1/9 (cohesin/dockerin = 1/1) was predicted to comprise cellulosome complexes displaying nine enzyme molecules^9^. The cellulosome complexes were reconstituted from the 3, 12, 30, and 40 components shown in Fig. 1. The top 3 components (the most abundant exoglucanase, Cel48S, the most abundant endoglucanase, Cel8A, and the major exoglucanase, Cel9K) were used for the reconstitution of the cellulosome complex in a previous study^9^. As shown in Fig. 2, which indicates the contents of the top 12 components in Fig. 1, the enzyme mixtures contained the cellulosomal components in close to the descending order of their reported NSAF values.

**Figure 3:**
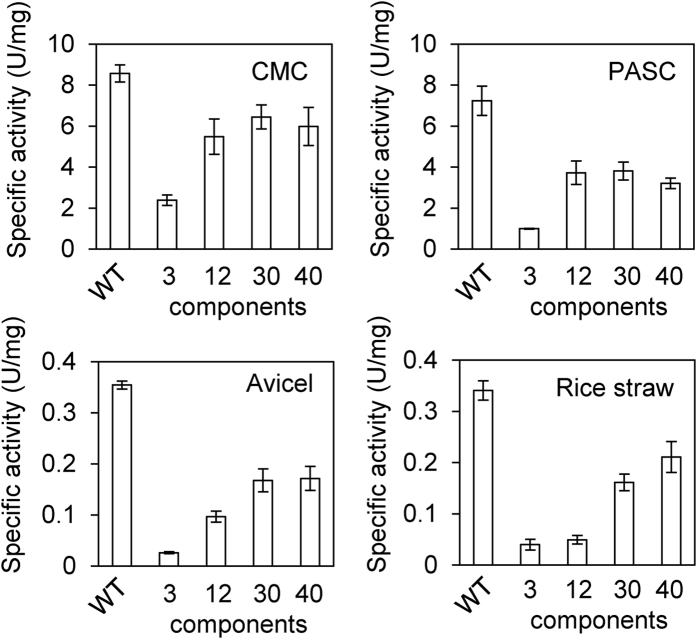
Enzymatic activities of the reconstituted cellulosomes for cellulosic substrates and delignified rice straw. The enzymatic activities were determined by measuring the amount of reducing sugars released from the substrate (present at 0.5%), as described in Methods. Cellulosome complexes were assembled by mixing scaffoldin and mixtures of 3, 12, 30, or 40 enzymes at a molar ratio of CipA/enzyme = 1/9 (cohesin/dockerin = 1/1). WT denotes native cellulosomes isolated from Avicel-grown cultures. The enzymatic activities for the cellulosic substrates, CMC, PASC, and Avicel, of the cellulosome complexes reconstituted from 3 components were measured previously^9^. Assays were performed at different concentrations of enzyme to determine if the amount of product increased in proportion to the amount of enzyme. Data are presented as the means from three to four independent experiments, and ±SE values are shown.

The activity of the reconstituted cellulosome towards cellulosic substrates with different degrees of crystallinity and towards delignified (acidified sodium chlorite + sodium bicarbonate-treated) rice straw was investigated and compared with the activity of the native cellulosomes isolated from Avicel-grown cultures (Fig. 3). The activity for carboxymethyl cellulose (CMC) and phosphoric acid-swollen cellulose (PASC) with a low crystallinity respectively increased by 2.3- and 3.7-fold when the number of cellulosomal components increased from 3 to 12, whereas a further increase in the number of components (from 12 to 40) did not substantially improve the cellulolytic activity of the cellulosome complex. In contrast, the activity for Avicel with a high crystallinity increased by 6.4-fold when the number of cellulosomal components increased from 3 to 30 components, whereas a further increase in the number of components (from 30 to 40) did not improve the cellulolytic activity. These results indicate that the degradation of crystalline cellulose requires greater enzymatic variety in the cellulosome complex than the degradation of amorphous cellulose. On the other hand, the activity for delignified rice straw was only marginally improved by increasing the number of cellulosomal components from 3 to 12, but increased 4.3-fold by increasing the number from 12 to 40. These results indicate that greater enzymatic variety in the cellulosome complex was more crucial for the degradation of delignified rice straw than for the degradation of crystalline cellulose. Furthermore, since the enzyme mixtures comprised of the 30 and 40 components were almost identical in the content of exo- and endo-glucanases, hemicellulase, and other protein (30-enzyme mix of 33% exoglucanase, 41% endoglucanase, 21% hemicellulase, and 5% other protein vs. 40-enzyme mix of 31% exoglucanase, 42% endoglucanase, 21% hemicellulase, and 4% other protein) (Fig. 2), these results suggest that the enzymatic diversity, rather than the enzymatic ratio, in the cellulosome complex was important for the degradation of delignified rice straw. In contrast, compared with the native cellulosome, the reconstituted cellulosome exhibiting the highest activity showed 75% of the activity for CMC but only ~50–60% of the native activity for PASC, Avicel, and delignified rice straw, suggesting that greater enzymatic variety (>40 components) may be required for the high activity exhibited by the native cellulosome or that the native cellulosome may have some mechanism for generating more synergy for the degradation of these substrates than *in vitro* reconstituted cellulosomes.

### Effect of Stoichiometric Assembly on Cellulosome Activity

**Figure 4:**
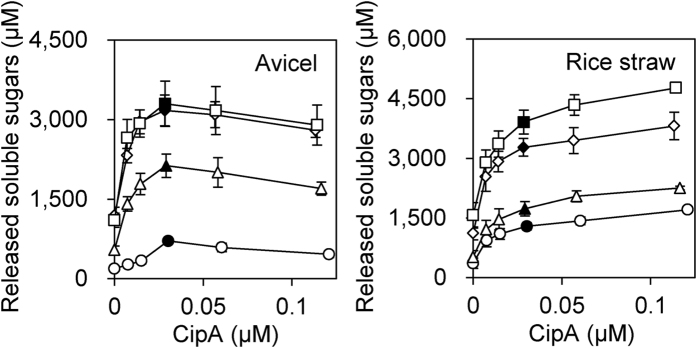
Activity profile of the reconstituted cellulosomes for crystalline cellulose and delignified rice straw. The enzymatic activities for Avicel and delignified rice straw of the cellulosome complexes reconstituted from 3 (circles), 12 (triangles), 30 (diamonds), and 40 (squares) components were determined by measuring the amount of reducing sugars released from 0.5% substrate at 55 °C for 24 h. Cellulosome complexes were assembled by mixing CipA at various concentrations with the enzyme mix at a fixed concentration (0.02 μg/μl). The activities of enzyme-saturated complexes assembled at a molar ratio of CipA/enzyme = 1/9 (cohesin/dockerin = 1/1) are shown by filled symbols. The enzymatic activity for Avicel of the cellulosome complexes reconstituted from 3 components was measured previously^9^. Data are presented as the means from three independent experiments, and ±SE values are shown.

To investigate the effect of the stoichiometry of cellulosome assembly on the activity for crystalline cellulose and plant biomass, we assembled cellulosome complexes by mixing different concentrations of full-length scaffoldin with fixed concentrations of the enzyme mixtures and then measured the activities for Avicel and delignified rice straw (Fig. 4, Table 1). The activity profile for the degradation of Avicel indicated that the reconstituted cellulosomes assembled at a molar ratio of CipA/enzyme = 1/9 (cohesin/dockerin = 1/1) exhibited the highest activity for Avicel regardless of the number of cellulosomal components used for the reconstitution of the cellulosome complex, which was consistent with the previous conclusion that stoichiometric assembly exhibited maximum synergy for the degradation of crystalline cellulose^9^. Moreover, the reconstituted cellulosome comprised of 40 components assembled at a molar ratio of CipA/enzyme = 1/9 (cohesin/dockerin = 1/1) showed 2.9-fold higher specific activity for Avicel than the unassembled enzyme mixture (Table 1), which was comparable to the previous result (4.0-fold synergy) observed for the cellulosome reconstituted using 3 components^9^. In contrast, surprisingly, the activity profile for the degradation of delignified rice straw indicated that cellulosomes reconstituted at a molar ratio of CipA/enzyme > 1/9 (cohesin/dockerin > 1/1) exhibited higher activity for delignified rice straw than cellulosomes reconstituted at a molar ratio of CipA/enzyme = 1/9 (cohesin/dockerin = 1/1), regardless of the number of cellulosomal components (Fig. 4), although the complexes assembled at CipA/enzyme > 1/9 (cohesin/dockerin > 1/1) were predicted to display fewer than nine enzyme molecules^9^. In fact, the reconstituted cellulosome comprised of 40 components assembled at a molar ratio of CipA/enzyme = 1/2.25 (cohesin/dockerin = 1/0.25) showed 1.3-fold higher specific activity for delignified rice straw than the cellulosomes reconstituted at a molar ratio of CipA/enzyme = 1/9 (cohesin/dockerin = 1/1) (Table 1). Therefore, these results indicate that a less complete enzyme complex displaying fewer than nine enzyme molecules was more efficient for the degradation of delignified rice straw than the enzyme-saturated complex, regardless of the enzymatic diversity in the cellulosome complex, although the enzyme-saturated complex exhibited maximum synergy for the degradation of crystalline cellulose.

## Discussion

Here, we synthesized 40 cellulosomal components of *C. thermocellum* ATCC 27405 using a wheat germ cell-free protein synthesis system (Supplementary Figs S1 and S2) and reconstituted supramolecular cellulosome complexes from full-length CipA and 3, 12, 30, or 40 components based on their contents in the native cellulosome isolated from cells grown on crystalline cellulose (Figs 1 and 2). The activities of these reconstituted cellulosomes toward cellulosic substrates and delignified rice straw indicated that greater enzymatic variety in the cellulosome complex generated more synergy for the degradation of crystalline cellulose and delignified rice straw, and that greater enzymatic variety in the cellulosome complex was more crucial for the degradation of delignified rice straw than for the degradation of crystalline cellulose (Fig. 3). This conclusion was consistent with the previous concept that greater enzymatic variety may lead to higher degradation activity for crystalline cellulose^9^. However, the cellulosome reconstituted from the 40 components showed only ~50–60% of the activities for PASC, Avicel, and delignified rice straw of the native cellulosome isolated from Avicel-grown cultures (Fig. 3). Because both the reconstituted and native cellulosomes had the enzymatic composition of the cellulosome isolated from cells grown on crystalline cellulose, it is unlikely that there is a substantial difference in the enzymatic composition between them. There are several possible explanations for the observed difference in enzymatic activity between the reconstituted and native cellulosomes. The genome of *C. thermocellum* ATCC 27405 contains ~70 cellulosomal genes encoding type-I dockerin-containing cellulosomal proteins, whereas the cellulosome reconstituted in this study contains only 40 components. While most of the remaining ~30 cellulosomal components are classified as hemicellulases, pectic enzymes, and proteins with unknown functions in the CAZy database are predicted to be the minor components of the *C. thermocellum* cellulosome^7^, it is likely that a wide variety of enzymes including various polysaccharide-degrading enzymes in the cellulosome complex contributes to the degradation of delignified rice straw, which contains various matrix polysaccharides. In addition, non-cellulosomal cellulases, such as Cel9I and Cel48Y^48^, in the cellulosome fraction after preparation by affinity digestion and cellulosomal proteins with unknown function may also play a crucial role in the high activities of the native cellulosome for crystalline cellulose and delignified rice straw. Moreover, because the wheat germ extract exhibits no detectable endogenous activity for various polysaccharides^49^, the contaminant proteins in the purified samples (Supplementary Fig. S1) are predicted not to be polysaccharide-degrading proteins, but their presence likely causes underestimation of the specific activity of the reconstituted cellulosomes. Furthermore, cellulosomal proteins prepared from *C. thermocellum* often contain O-linked glycosylation, mainly at the Thr/Pro-rich linker regions^50^. This suggests that the loss of such native modifications may also contribute to the lower specific activity of the reconstituted cellulosomes. Alternatively, it is also possible that the native cellulosome has some mechanism for generating more synergy for the degradation of crystalline cellulose and plant biomass than *in vitro* reconstituted cellulosomes. For example, electron microscopic studies have revealed that native *C. thermocellum* cellulosomes isolated from cells grown on crystalline cellulose contain various types of loosely or tightly packed complexes with a somewhat ordered ultrastructure^51^. These findings suggest that the native cellulosome may not necessarily be comprised only of enzyme-saturated complexes but also of less complete complexes displaying fewer than nine enzyme molecules, and that the cellulosomal components may not be located in a completely random manner but in a somewhat ordered manner in the native cellulosome. Although it remains unknown whether the ultrastructure observed for the native cellulosome contributes to cellulosome activity, the activity profile for degradation of delignified rice straw showed the surprising results that an incompletely enzyme-saturated complex was more efficient for the degradation of delignified rice straw than an enzyme-saturated complex regardless of the enzymatic diversity in the cellulosome complex (Fig. 4, Table 1). This is despite the fact that the enzyme-saturated complex exhibited the greatest synergy for the degradation of crystalline cellulose (Fig. 4, Table 1)^9^. Therefore, these findings suggest that efficient degradation of different substrates by the cellulosome may require not only a different enzymatic composition, but also different cellulosome structures. Thus, it will be interesting to investigate whether the cellulosomal structures found in the native cellulosome contribute to cellulosome activity. Further comparative studies on *in vitro* reconstituted and native cellulosomes may provide clues for solving this cellulosome activity paradox.

## Methods

### Materials

*C. thermocellum* ATCC 27405 (NBRC 103400) was obtained from the National Institute of Technology and Evaluation, Japan and grown at 55 °C using M medium^52^ containing 0.5% Avicel (Avicel PH-101; Sigma-Aldrich, Japan) as a carbon source. The nucleotide sequence of *C. thermocellum* genomic DNA is available from the *National* Center for Biotechnology Information database under accession no. NC_009012.1. Plasmids pEUGST-GFP and pMWGST-GFP, respectively used as a high- and low-copy number cassette vector for the construction of pEU and pMW derivatives for the cell-free protein synthesis of GST fusion proteins, were previously constructed^9^,^12^. The cellulosomal enzymes Cel8A, Cel9,44J, Cel9K, and Cel48S and the scaffoldin and miniscaffoldin proteins, CipA and ΔCipA, respectively, were purified as described previously^9^,^12^, and wheat germ extract was prepared as reported previously^53^. *E. coli* DH5α (Takara, Japan) was used as a cloning host. All PCR primers were synthesized by Fasmac Co., Ltd., Japan and are listed in Supplementary Table S1. The nucleotide sequences of pEUGST-NPr3′, pEU-C5′, pEUUn, and 2pEUDn were described previously^9^.

### DNA Substrates for Cell-Free Protein Synthesis of GST Fusion Proteins

Plasmid pEU derivatives for synthesizing GST fusion cellulosomal components, except for GST fusion Cbh9A, were constructed as follows. The cellulosomal component genes were PCR-amplified from genomic DNA prepared from *C. thermocellum* ATCC 27405 cells using the primers following the nomenclature “gene product name”-NPr5′ and “gene product name”-CF3′ listed in Supplementary Table S1. A plasmid DNA fragment corresponding to pEUGST was PCR-amplified from pEUGST-GFP using primers pEUGST-NPr3′ and pEU-C5′. Each cellulase gene and plasmid DNA fragment was ligated using an In-fusion HD Cloning Kit (Clontech, Japan) to yield plasmids for cell-free synthesis of the GST-fusion proteins, which were then introduced into DH5α cells. Positive transformants were selected on Luria-Bertani (LB) agar plates containing 50 μg/ml ampicillin and incubated at 37 °C. The Cbh9A gene was cloned into plasmid pMWGST-GFP, a low-copy number cassette vector. The Cbh9A gene amplified from genomic DNA using primers Cbh9A-NPr5′ and Cbh9A-CF3′ and the plasmid DNA fragment amplified from pMWGST-GFP using primers pEUGST-NPr3′ and pEU-C5′ were ligated using an In-fusion HD Cloning Kit and then introduced into DH5α cells. Positive transformants were selected on LB agar plates containing 30 μg/ml kanamycin and incubated at 30 °C. The DNA substrates for cell-free protein synthesis of the GST fusion proteins were PCR-amplified from pEUGST and pMWGST fusion derivatives using primers pEUUn and 2pEUDn. PCR was performed for 25 cycles using the following conditions: 96 °C for 30 s, 50 or 55 °C for 30 s, and 72 °C for 60 s/kbp with PrimeSTAR HS or GXL DNA polymerase (Takara) using a Takara PCR Thermal Cycler Dice TP-650. The cellulosomal genes in the pEU and pMW derivatives were sequenced by the dideoxy chain termination method with fluorescent dye terminators (Eurofins Genomics, Japan).

### Cell-free Protein Synthesis and Purification

Wheat germ cell-free protein synthesis and purification of the GST fusion proteins were performed as described previously^54^. The synthesized fusion proteins were cleaved with PreScission protease in a glutathione-Sepharose 4B MicroSpin column (GE Healthcare, Japan). The flow-through fraction contained proteins of the predicted sizes for FLAG tag-fused mature protein, as revealed by SDS-PAGE on 4–20% gradient gels (ATTO, Japan), staining with Coomassie brilliant blue, and western blot analysis using anti-FLAG M2 monoclonal antibody (Sigma-Aldrich) with an ECL Select detection kit (GE Healthcare) as described previously^9^. The protein concentration was estimated by densitometric analysis with ImageJ software (National Institutes of Health, USA) using bovine serum albumin (BSA) as a standard.

### Cellulosome Reconstitution

Cellulosome complexes were assembled by mixing enzyme mixtures at a fixed concentration (0.02 μg/μl) and CipA of various concentrations in buffer A (50 mM sodium acetate pH 5.5, 2 mM CaCl_2_, 2 mM dithiothreitol (DTT), and 0.01% BSA) at 40 °C for 1 h. The binding of each cellulosomal component to scaffoldin was analyzed by an electrophoretic mobility shift assay of miniscaffoldin (ΔCipA) using native PAGE on 4–20% gradient gels with western blot analysis using an anti-Strep tag monoclonal antibody (THE NWSHPQFEK Tag antibody; GenScript, USA) as described previously^9^. To assay cellulosome activity, cellulosome complexes were assembled by mixing different concentrations of full-length scaffoldin and fixed concentrations of the enzyme mixture prepared at the reported ratios of the NSAF values of each cellulosomal component in the native cellulosome prepared from cells grown on crystalline cellulose (Supplementary Fig. S1)^7^.

### Cellulosome Isolation

Native cellulosomes were isolated from the supernatant of Avicel-grown *C. thermocellum* ATCC 27405 cultures using the affinity digestion method^55^. Briefly, supernatants of Avicel-grown cultures were incubated with 0.01% PASC overnight at 4 °C. Cellulosome-bound PASC was resuspended in a reaction buffer containing 25 mM sodium acetate pH 5.5, 10 mM CaCl_2_, 10 mM L-cysteine, 2 mM ethylenediaminetetraacetic acid (EDTA), and 2 mM DTT and dialyzed against the reaction buffer at 55 °C in a cellulose ester membrane (Spectrum Laboratories, Inc., USA) until the PASC dissolved (~6 h), then dialyzed against a storage buffer containing 25 mM Tris-HCl pH 7.0, 10 mM CaCl_2_, and 2 mM DTT overnight at 4 °C for buffer exchange. The protein concentration was determined with the Bradford assay^56^ using BSA as a standard.

### Delignification of Rice Straw

Rice straw was delignified using acidified sodium chlorite and sodium bicarbonate in accordance with reported procedures^57^ with slight modifications. Rice straw was ground using a laboratory cutting mill to a particle size of 0.5–1.0 mm and then delignified in deionized water (60 ml/g biomass) using sodium chlorite (0.4 g/g biomass) and glacial acetic acid (0.08 ml/g biomass) at 70 °C for 1 h. The treated rice straw was immersed in 0.5% sodium bicarbonate solution overnight at room temperature and then autoclaved at 121 °C for 15 min. After washing the rice straw using ethanol and acetone, the sample was dried at 105 °C for 2 h. The chemical composition of rice straw treated with acidified sodium chlorite and sodium bicarbonate was 2% lignin, 20% pentose, and 78% hexose; the lignin content was determined by the Klason lignin method in the Laboratory Analytical Procedures provided by the National Renewable Energy Laboratory^58^, and the pentose and hexose contents were determined using orcinol and anthrone reagents, respectively, as described previously^59^,^60^.

### Enzyme Assay

Activities of the cellulosomal enzymes and the reconstituted cellulosomes were assayed at 55 °C in buffer A, as described previously^9^,^12^. Assay substrates were used at a final concentration of 5 mg/ml (0.5%) and included: Avicel, PASC prepared from Avicel as described previously^61^, CMC (Sigma-Aldrich), xylan from beech wood (Tokyo Chemical Industry, Japan), arabinoxylan from wheat flour (Megazyme, Ireland), xyloglucan from tamarind (Megazyme), mannan from carob (Megazyme), lichenan from Icelandic moss (Megazyme), galactan from potato (Megazyme), and delignified (acidified sodium chlorite + sodium bicarbonate-treated) rice straw. The amount of reducing sugars released from the substrate was quantified using 3′,5′-dinitrosalicylic acid reagent^62^ by measuring the absorbance at 535 nm using glucose, xylose, or galactose as a standard. To assay rhamnogalacturonan lyase activity, pectin from apple (Sigma-Aldrich) was used as a substrate at a final concentration of 5 mg/ml (0.5%). The concentration of unsaturated products was quantified by measuring the absorbance at 235 nm, and the molar coefficient used for the unsaturated product at 235 nm was 4,600 M^−1^cm^−1 ^43. To assay chitinase and rhamnogalacturonan acetyl esterase activities, *p*-nitrophenyl *β*-D-N,N′,N′′-triacetylchitotriose (pNP-(GlcNAc)_3_) (Sigma-Aldrich) and *p*-nitrophenyl acetate (pNPA) (Tokyo Chemical Industry) were respectively used as substrate at a final concentration of 0.5 and 0.1 mM^45^,^63^. Rhamnogalacturonan acetyl esterase activity was assayed in buffer A containing 50 mM sodium citrate, pH 5.5, instead of 50 mM sodium acetate, pH 5.5. After completion of the incubation period, 100 mM sodium carbonate solution was added to the reaction mixture and the concentration of *p*-nitrophenol was quantified by measuring the absorbance at 420 nm. To assay activity towards recalcitrant substrates, the incubation time was extended from 30 min to 24 h. One unit of enzymatic activity was defined as the amount of enzyme producing 1 μmol of reaction product per minute, and specific activity was defined as the enzymatic activity per mg of enzyme. Assays were performed at different concentrations of enzyme to determine if the amount of product increased in proportion to the amount of enzyme.

## Additional Information

**How to cite this article**: Hirano, K. *et al*. Enzymatic diversity of the *Clostridium thermocellum* cellulosome is crucial for the degradation of crystalline cellulose and plant biomass. *Sci. Rep.* **6**, 35709; doi: 10.1038/srep35709 (2016).

## Supplementary Material

Supplementary Information

## Figures and Tables

**Table 1:** Enzymatic activities of reconstituted cellulosomes for crystalline cellulose and delignified rice straw.

CipA/enzyme^b^	Specific activity (U/mg)^a^			
	Avicel		Rice straw	
0	0.058 ± 0.0061	(1.0)^c^	0.090 ± 0.010	(1.0)^c^
1/9	0.17 ± 0.024	(2.9)^c^	0.21 ± 0.030	(2.3)^c^
1/2.25	0.15 ± 0.016	(2.7)^c^	0.27 ± 0.015	(3.0)^c^

^a^Cellulosome complexes were assembled by mixing CipA at various concentrations and the 40 enzyme mix at a fixed concentration. Assays were performed at different concentrations of enzyme to determine if the amount of product increased in proportion to the amount of enzyme. Data are presented as the means from three independent experiments, and ±SE are shown.

^b^The values of CipA/enzyme indicate the molar ratio of the scaffoldin protein relative to the enzyme mix.

^c^The relative activities of the cellulosome complexes to the unassembled enzyme mixture are indicated in parentheses.
