# Supplementary material for: Enzymatic diversity of the Clostridium thermocellum cellulosome is crucial for the degradation of crystalline cellulose and plant biomass
Source: Sci Rep. 2016 Oct 19;6:35709. doi: 10.1038/srep35709 (PMC5069625; doi:10.1038/srep35709)
Supplement: Supplementary Information [file srep35709-s1.pdf]

Enzymatic diversity of the *Clostridium thermocellum* cellulosome is crucial for the degradation of crystalline cellulose and plant biomass.

Katsuaki Hirano[a], Masahiro Kurosaki[a], Satoshi Nihei[a], Hiroki Hasegawa[a], Suguru Shinoda[a], Mitsuru Haruki[a], and Nobutaka Hirano[a]

[a]*Department of Chemical Biology & Applied Chemistry, College of Engineering, Nihon University, Koriyama, Fukushima 963-8642, Japan*

Supplementary figure legends

Fig. S1. SDS-PAGE analysis of the purified cellulosomal components.

The 40 purified cellulosomal components were separated by SDS-PAGE on a 4-20% gel and stained with Coomassie brilliant blue.

Fig. S2. Western blot of the purified cellulosomal components.

The 40 purified cellulosomal components were detected by western blotting with anti-FLAG M2 monoclonal antibody targeting the C-terminal FLAG tag of the cellulosomal components.

Fig. S3. Electrophoretic mobility shift assay of the binding of each cellulosomal component to the scaffoldin protein.

The binding of each cellulosomal component to scaffoldin was analyzed by an electrophoretic mobility shift assay of miniscaffoldin (ΔCipA), which contains two cohesin modules and a CBM3a, as described in Materials and Methods. Minicellulosome complexes assembled at a molar ratio of ΔCipA/enzyme = ~1/2 to 1/4 (cohesin/dockerin = ~1/1 to 1/2) were separated by native PAGE on 4-20% gradient gels and then detected by western blotting using an anti-Strep tag monoclonal antibody. The bands corresponding to ΔCipA are indicated. Binding of Cel48S, Cel8A, and Cel9K to scaffoldin was confirmed previously (9).

Fig. S4. Substrate specificities of the cellulosomal enzymes.

Substrate specificities of the cellulosomal enzymes were analyzed at 55 °C, as described in Materials and Methods. The specific activities for CMC and PASC of Cel48S, Cel8A, and Cel9K were measured previously (9). "—" means activity was not determined. Assays were performed at different concentrations of enzyme to determine if the amount of product increased in proportion to the amount of enzyme.

Figure S1

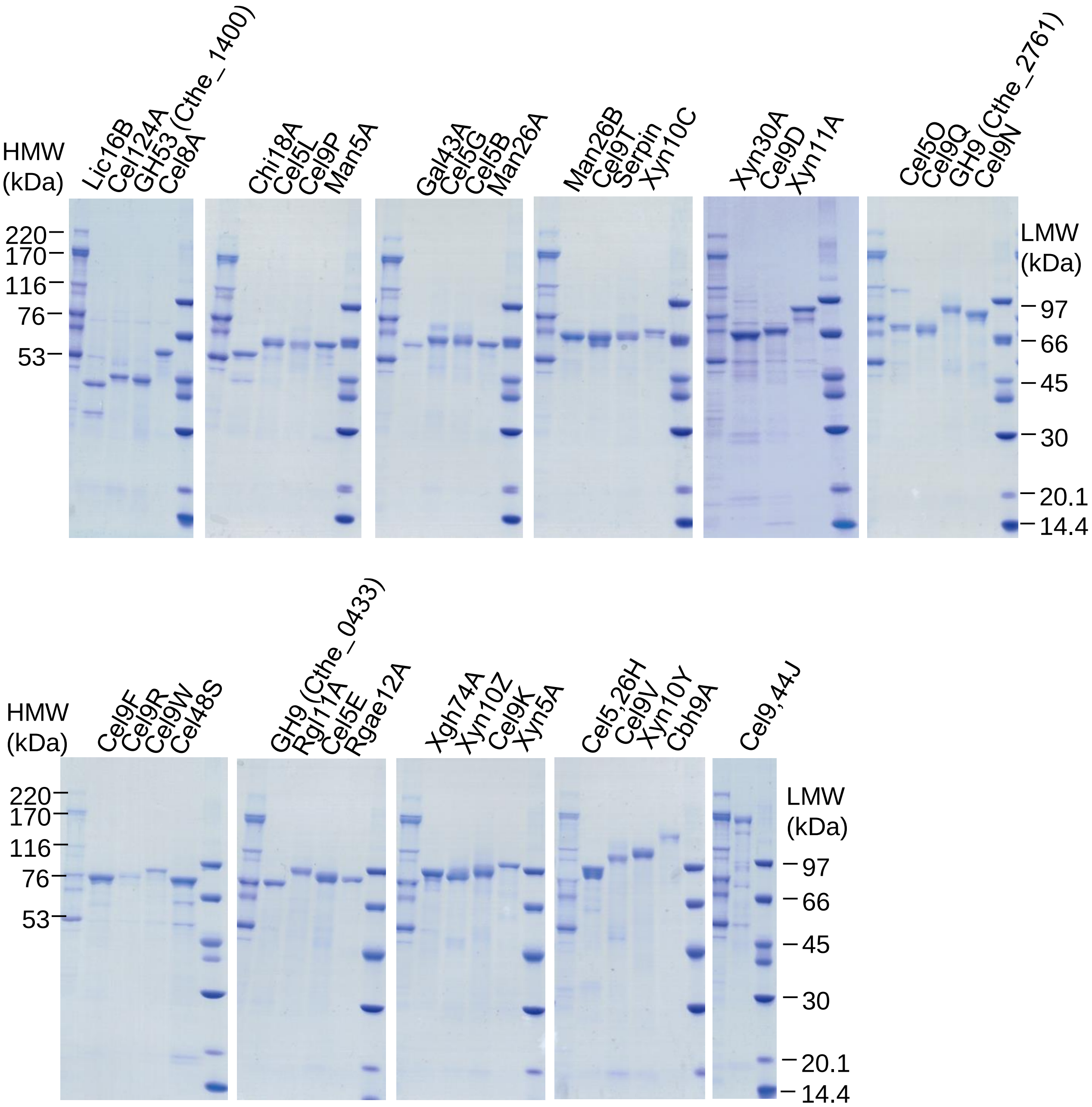
HMW (kDa)
220
170
116
76
53
Lic16B
Cel124A
GH53 (Cthe_1400)
Cel8A
Chi18A
Cel5L
Cel9P
Man5A
Gal43A
Cel5G
Cel5B
Man26A
Man26B
Cel9T
Serpin
Xyn10C
Xyn30A
Cel9D
Xyn11A
Cel5O
Cel9Q
GH9 (Cthe_2761)
Cel9N
LMW (kDa)
97
66
45
30
20.1
14.4
HMW (kDa)
220
170
116
76
53
Cel9F
Cel9R
Cel9W
Cel48S
GH9 (Cthe_0433)
Rgl11A
Cel5E
Rgae12A
Xgh74A
Xyn10Z
Cel9K
Xyn5A
Cel5,26H
Cel9V
Xyn10Y
Cbh9A
Cel9,44J
LMW (kDa)
97
66
45
30
20.1
14.4

Figure S2

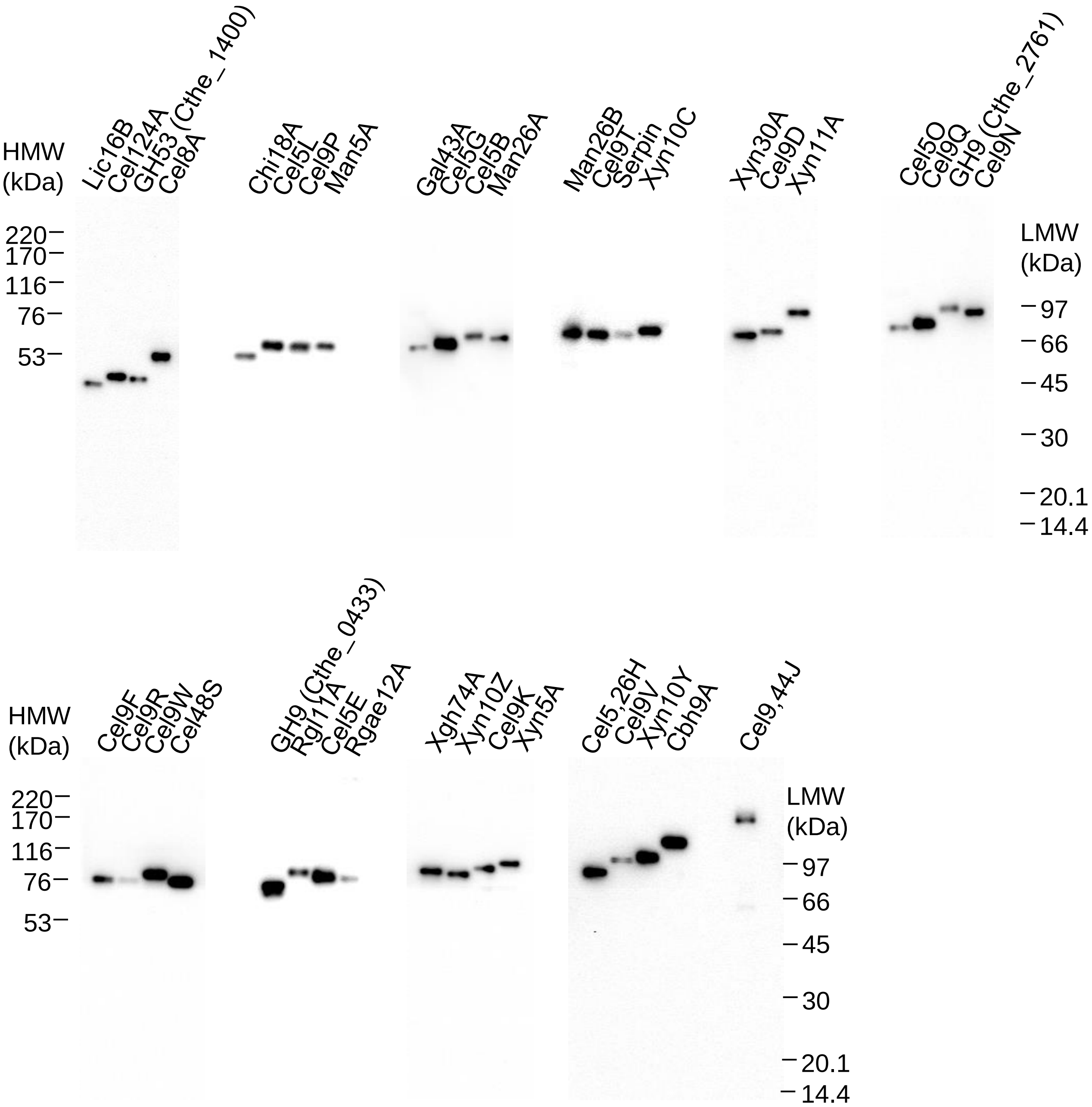
HMW (kDa)
220
170
116
76
53
Lic16B
Cel124A
GH53 (Cthe_1400)
Cel8A
Chi18A
Cel5L
Cel9P
Man5A
Gal43A
Cel5G
Cel5B
Man26A
Man26B
Cel9T
Serpin
Xyn10C
Xyn30A
Cel9D
Xyn11A
Cel5O
Cel9Q
GH9 (Cthe_2761)
Cel9N
LMW (kDa)
97
66
45
30
20.1
14.4
HMW (kDa)
220
170
116
76
53
Cel9F
Cel9R
Cel9W
Cel48S
GH9 (Cthe_0433)
Rgl11A
Cel5E
Rgae12A
Xgh74A
Xyn10Z
Cel9K
Xyn5A
Cel5,26H
Cel9V
Xyn10Y
Cbh9A
Cel9,44J
LMW (kDa)
97
66
45
30
20.1
14.4

Figure S3

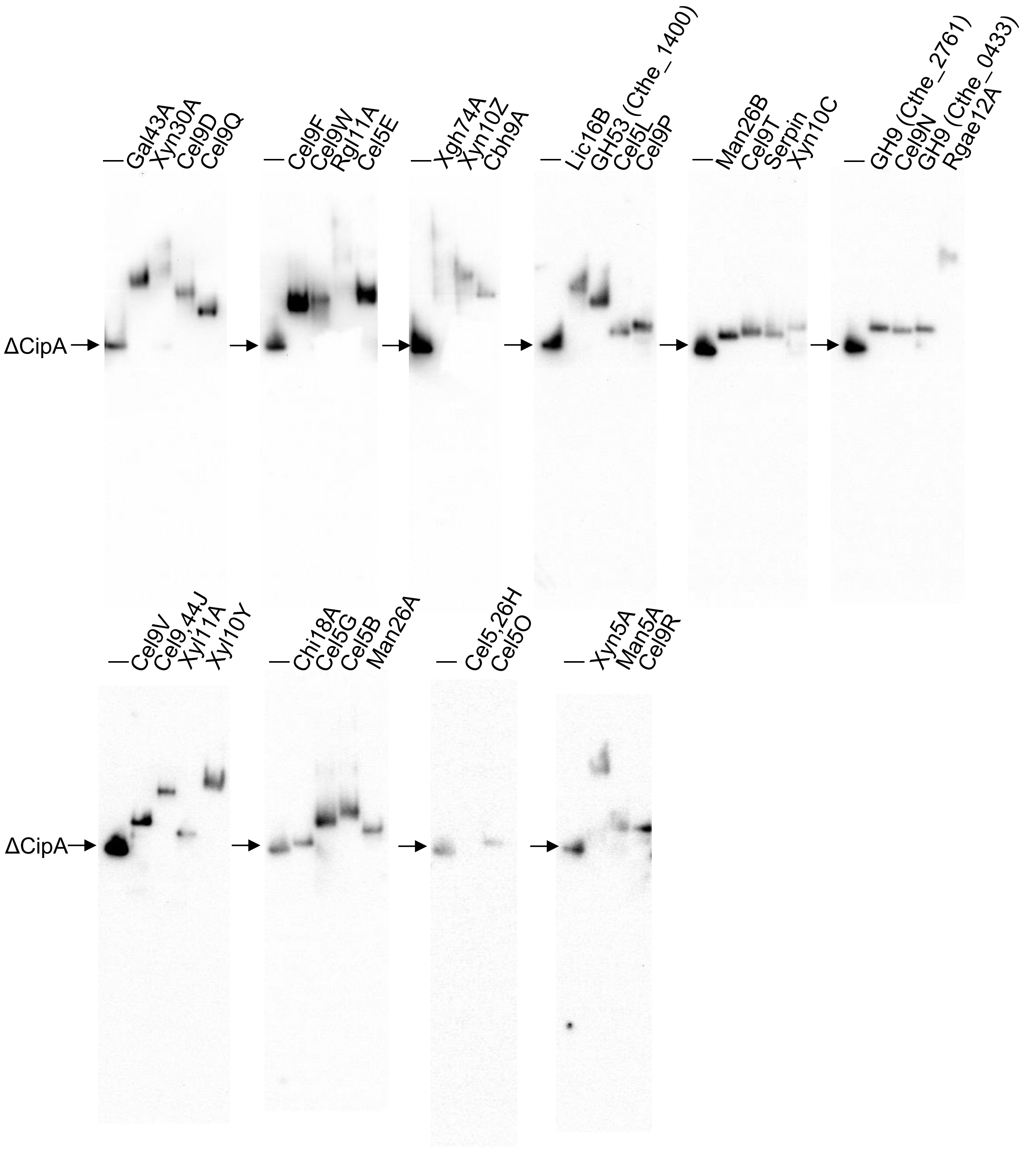
— Gal43A Xyn30A Cel9D Cel9Q
— Cel9F Cel9W Rgl11A Cel5E
— Xgh74A Xyn10Z Cbh9A
— Lic16B GH53 (Cthe_1400) Cel5L Cel9P
— Man26B Cel9T Serpin Xyn10C
— GH9 (Cthe_2761) Cel9N GH9 (Cthe_0433) Rgae12A
ΔCipA
— Cel9V Cel9,44J Xyl11A Xyl10Y
— Chi18A Cel5G Cel5B Man26A
— Cel5,26H Cel5O
— Xyn5A Man5A Cel9R
ΔCipA

Figure S4

Gene product	Locus tag	NSAF	Specific activity (U/mg)											Putative function or Experimentally confirmed activity
			CMC	PASC	Xylan	Arabinoxylan	Mannan	Xyloglucan	Lichenan	Galactan	pNP-(GlcNAc)$_3$	pNPA	Pectin	
Cel48S	Cthe_2089	4.06	0.0031	0.087	—	—	—	—	—	—	—	—	—	exo-β-1,4-glucanase releasing cellobiose from the reducing end
Cel8A	Cthe_0269	1.82	14	3.7	—	—	—	—	—	—	—	—	—	endo-β-1,4-glucanase
Cel9K	Cthe_0412	0.72	0.030	0.37	—	—	—	—	—	—	—	—	—	exo-β-1,4-glucanase releasing cellobiose from the non-reducing end
Xyn11A	Cthe_2972	1.13	—	—	42	—	—	—	—	—	—	—	—	acetylxylan esterase
Man5A	Cthe_0821	0.92	—	—	—	—	49	—	—	—	—	—	—	β-mannanase
Serpin	Cthe_0190	0.67	—	—	—	—	—	—	—	—	—	—	—	serine protease inhibitor
Cel9Q	Cthe_0625	0.62	3.6	3.3	—	—	—	—	—	—	—	—	—	endo-β-1,4-glucanase
Cbh9A	Cthe_0413	0.59	0.33	1.2	—	—	—	—	—	—	—	—	—	exo-β-1,4-glucanase releasing cellobiose from the non-reducing end
Cel9F	Cthe_0543	0.55	16	7.0	—	—	—	—	—	—	—	—	—	endo-β-1,4-glucanase
Cel5B	Cthe_0536	0.54	6.3	7.1	—	—	—	—	—	—	—	—	—	endo-β-1,4-glucanase
Cel9T	Cthe_2812	0.44	8.4	2.8	—	—	—	—	—	—	—	—	—	endo-β-1,4-glucanase
Cel9R	Cthe_0578	0.43	3.9	7.0	—	—	—	—	—	—	—	—	—	endo-β-1,4-glucanase
Man26A	Cthe_2811	0.47	—	—	—	—	44	—	—	—	—	—	—	β-mannanase
Cel5G	Cthe_2872	0.42	13	8.4	—	—	—	—	—	—	—	—	—	endo-β-1,4-glucanase
Cel5E	Cthe_0797	0.42	24	2.3	1.8	—	0.89	—	—	—	—	—	—	endo-β-1,4-glucanase, endo-β-1,4-xylanase, and β-mannanase
Xgh74A	Cthe_1398	0.41	—	—	—	—	—	22	—	—	—	—	—	xyloglucanase
Cel9,44J	Cthe_0624	0.41	21	0.65	1.4	—	—	45	—	—	—	—	—	endo-β-1,4-glucanase, endo-β-1,4-xylanase, and xyloglucanase
Xyn10C	Cthe_1838	0.35	—	—	9.8	—	—	—	—	—	—	—	—	endo-β-1,4-xylanase
Cel9W	Cthe_0745	0.30	5.9	3.8	—	—	—	—	—	—	—	—	—	β-1,4-glucanase
Cel9P	Cthe_0274	0.24	2.6	3.1	—	—	—	—	—	—	—	—	—	endo-β-1,4-glucanase
Cel9N	Cthe_0043	0.23	2.9	0.32	—	—	—	—	—	—	—	—	—	endo-β-1,4-glucanase
Cel5L	Cthe_0405	0.15	9.7	8.6	—	—	—	—	—	—	—	—	—	β-1,4-glucanase
Cel9D	Cthe_0543	0.13	19	7.2	—	—	—	—	—	—	—	—	—	endo-β-1,4-glucanase
Xyn10Z	Cthe_1963	0.13	—	—	37	—	—	—	—	—	—	—	—	endo-β-1,4-xylanase
Cel9V	Cthe_2760	0.12	0.33	0.21	—	—	—	—	—	—	—	—	—	β-1,4-glucanase
Cel5O	Cthe_2147	0.10	7.1	2.0	—	—	—	—	—	—	—	—	—	exo-β-1,4-glucanase releasing cellobiose from the reducing end
Lic16B	Cthe_0211	0.07	—	—	—	—	—	—	480	—	—	—	—	lichenase
Xyn10Y	Cthe_0912	0.04	—	—	31	—	—	—	—	—	—	—	—	endo-β-1,4-xylanase
Chi18A	Cthe_0270	0.03	—	—	—	—	—	—	—	—	3.1	—	—	chitinase
Cel5,26H	Cthe_1472	0.02	15	3.0	1.1	—	—	—	—	—	—	—	—	endo-β-1,4-glucanase and endo-β-1,4-xylanase
Cel124A	Cthe_0435	0.19	0.47	0.17	—	—	—	—	—	—	—	—	—	endo-β-1,4-glucanase
GH9 (Cthe_2761)	Cthe_2761	0.15	6.1	1.7	—	—	—	—	—	—	—	—	—	β-1,4-glucanase
GH9 (Cthe_0433)	Cthe_0433	0.15	6.7	1.5	—	—	—	—	—	—	—	—	—	β-1,4-glucanase
Man26B	Cthe_0032	0.08	—	—	—	—	33	—	—	—	—	—	—	β-mannanase
GH53 (Cthe_1400)	Cthe_1400	0.07	—	—	—	—	—	—	—	1400	—	—	—	endo-β-1,4-galactanase
Gal43A	Cthe_0661	0.07	—	—	—	—	—	—	—	<0.10	—	—	—	exo-β-1,3-galactanase
Rgl11A	Cthe_0246	0.07	—	—	—	—	—	—	—	—	—	—	0.30	rhamnogalacturonan lyase
Rgae12A	Cthe_3141	0.04	—	—	—	—	—	—	—	—	—	0.048	—	rhamnogalacturonan acetyl esterase
Xyn5A	Cthe_2193	0.04	—	—	—	6.1	—	—	—	—	—	—	—	arabinoxylan-specific xylanase
Xyn30A	Cthe_3012	0.03	—	—	0.52	—	—	—	—	—	—	—	—	glucuronoxylan xylanohydrolase

Table S1. Primers used for DNA amplification.

Name	Nucleotide sequence
Xyn11A-NPr5′	5′-TTCCAAGGTCCACTGGGATCCACTAGTGATGTAGTAATTACGTCAAACC-3′
Xyn11A-CF3′	5′-TGGGACGTCGACGCGTTTACTTGTCATCGTCATCCTTGTAGTCGTCACCCGGTACAGAGTTATACATTC-3′
Man5A-NPr5′	5′-TTCCAAGGTCCACTGGGATCCACTAGTGCGGATGACATTTATCCGGGACTTAG-3′
Man5A-CF3′	5′-TGGGACGTCGACGCGTTTACTTGTCATCGTCATCCTTGTAGTCGTCACCTGGTGTCACTATTCTCGTAT-3′
Serpin-NPr5′	5′-TTCCAAGGTCCACTGGGATCCACTAGTGACGGAAACTGGAAAACTTATTACG-3′
Serpin-CF3′	5′-TGGGACGTCGACGCGTTTACTTGTCATCGTCATCCTTGTAGTCGTCACCATTAATCAACCCGCCGTCAC-3′
Cel9Q-NPr5′	5′-TTCCAAGGTCCACTGGGATCCACTAGTGCAGGAAGCTATAACTATGCGGAAG-3′
Cel9Q-CF3′	5′-TGGGACGTCGACGCGTTTACTTGTCATCGTCATCCTTGTAGTCGTCACCTTCTACCGGAAATTTATCTA-3′
Cbh9A-NPr5′	5′-TTCCAAGGTCCACTGGGATCCACTAGTTTAGAAGATAATTCTTCGACTTTG-3′
Cbh9A-CF3′	5′-TGGGACGTCGACGCGTTTACTTGTCATCGTCATCCTTGTAGTCGTCACCTCGATATGGCAATTCTTCTA-3′
Cel9F-NPr5′	5′-TTCCAAGGTCCACTGGGATCCACTAGTGCGGATTTCAACTATGGTGAGG-3′
Cel9F-CF3′	5′-GGGACGTCGACGCGTTTACTTGTCATCGTCATCCTTGTAGTCGTCACCCTGTTCAGCCGGGAATTTTTC-3′
Cel5B-NPr5′	5′-TTCCAAGGTCCACTGGGATCCACTAGTGCAGAAGGGTCATATGCTGATTTG-3′
Cel5B-CF3′	5′-TGGGACGTCGACGCGTTTACTTGTCATCGTCATCCTTGTAGTCGTCACCTTTATACGGCAACTCACTTA-3′
Cel9T-NPr5′	5′-TTCCAAGGTCCACTGGGATCCACTAGTGGAGAATACAATTATGCAAAGG-3′
Cel9T-CF3′	5′-TGGGACGTCGACGCGTTTACTTGTCATCGTCATCCTTGTAGTCGTCACCTATAGGGAGAGACGGTATGC-3′
Cel9R-NPr5′	5′-TTCCAAGGTCCACTGGGATCCACTAGTGCAGACTATAACTATGGAGAAGC-3′
Cel9R-CF3′	5′-TGGGACGTCGACGCGTTTACTTGTCATCGTCATCCTTGTAGTCGTCACCTGAATTTCCGGGTATGGTTG-3′
Man26A-NPr5′	5′-TTCCAAGGTCCACTGGGATCCACTAGTGTGCTTTCTGACGGGGATAAGTATG-3′
Man26A-CF3′	5′-GGACGTCGACGCGTTTACTTGTCATCGTCATCCTTGTAGTCGTCACCCTGTTGTTCAACGGGAAAACTC-3′
Cel5G-NPr5′	5′-TTCCAAGGTCCACTGGGATCCACTAGTCAGAATACCGGTTCAACAGCTAC-3′
Cel5G-CF3′	5′-GGGACGTCGACGCGTTTACTTGTCATCGTCATCCTTGTAGTCGTCACCGGTGGTGTGCGGCAGTTTGTC-3′
Cel5E-NPr5′	5′-TTCCAAGGTCCACTGGGATCCACTAGTGCATCACCGGTAAAAGGCTTTCAG-3′
Cel5E-CF3′	5′-ACGTCGACGCGTTTACTTGTCATCGTCATCCTTGTAGTCGTCACCCCATCCAAGCTTGTTTTTATTTC-3′

Xgh74A-NPr5′	5′-TTCCAAGGTCCACTGGGATCCACTAGTGTAACCAGCGTGCCTTACAAATG-3′
Xgh74A-CF3′	5′-TGGGACGTCGACGCGTTTACTTGTCATCGTCATCCTTGTAGTCGTCACCTTTTTTCGGCAGCTCCGGAA-3′
Xyn10C-NPr5′	5′-TTCCAAGGTCCACTGGGATCCACTAGTGCAGCTCTGATTTACGATGATTTTG-3′
Xyn10C-CF3′	5′-TGGGACGTCGACGCGTTTACTTGTCATCGTCATCCTTGTAGTCGTCACCAAGTTCTCTCAGAACGAGTT-3′
Cel9W-NPr5′	5′-TTCCAAGGTCCACTGGGATCCACTAGTTCTGCAGCAACTACATTCAACTAC-3′
Cel9W-CF3′	5′-TGGGACGTCGACGCGTTTACTTGTCATCGTCATCCTTGTAGTCGTCACCAGGTGCGTAAGGCAGTTTGC-3′
Cel9P-NPr5′	5′-TTCCAAGGTCCACTGGGATCCACTAGTGATTATGCCACCGCATTAAAATAC-3′
Cel9P-CF3′	5′-GACGTCGACGCGTTTACTTGTCATCGTCATCCTTGTAGTCGTCACCATTTATGATGTTTCCATAGATAT-3′
Cel9N-NPr5′	5′-TTCCAAGGTCCACTGGGATCCACTAGTGCTTCTTCGCCTCGTTACGGCGGTG-3′
Cel9N-CF3′	5′-GGACGTCGACGCGTTTACTTGTCATCGTCATCCTTGTAGTCGTCACCTATAGGTAACGAACCAATTAAC-3′
Cel5L-NPr5′	5′-TTCCAAGGTCCACTGGGATCCACTAGTGCCGATCCGAACAATGACGAC-3′
Cel5L-CF3′	5′-GACGTCGACGCGTTTACTTGTCATCGTCATCCTTGTAGTCGTCACCTATTGGTATTTTAAGCACTTTCC-3′
Cel9D-NPr5′	5′-TTCCAAGGTCCACTGGGATCCACTAGTATTGAGACCAAAGTGTCAGCTG-3′
Cel9D-CF3′	5′-GGACGTCGACGCGTTTACTTGTCATCGTCATCCTTGTAGTCGTCACCTATTGGTAATTTCTCGATTACC-3′
Xyn10Z-NPr5′	5′-TTCCAAGGTCCACTGGGATCCACTAGTGCATCCTTGCCAACCATGCCG-3′
Xyn10Z-CF3′	5′-TGGGACGTCGACGCGTTTACTTGTCATCGTCATCCTTGTAGTCGTCACCATAGCCCATAAGAGCTTCC-3′
Cel9V-NPr5′	5′-TTCCAAGGTCCACTGGGATCCACTAGTGAAGCACCACCTGCGACTTTTAC-3′
Cel9V-CF3′	5′-GACGTCGACGCGTTTACTTGTCATCGTCATCCTTGTAGTCGTCACCAAAAGACGTTATTATGCCAAGAA-3′
Cel5O-NPr5′	5′-TTCCAAGGTCCACTGGGATCCACTAGTGATGACACTTCTGAAGAACCCGC-3′
Cel5O-CF3′	5′-GACGTCGACGCGTTTACTTGTCATCGTCATCCTTGTAGTCGTCACCTTCATTTTGGTTTTCTTCCACCG-3′
Lic16B-NPr5′	5′-TTCCAAGGTCCACTGGGATCCACTAGTGAAGCCGCAACTGTGGTAAATACG-3′
Lic16B-CF3′	5′-GGACGTCGACGCGTTTACTTGTCATCGTCATCCTTGTAGTCGTCACCAAGTGACGGAATTGCCCGTATC-3′
Xyn10Y-NPr5′	5′-TTCCAAGGTCCACTGGGATCCACTAGTGATTATGAAGTGGTTCATGACAC-3′
Xyn10Y-CF3′	5′-GACGTCGACGCGTTTACTTGTCATCGTCATCCTTGTAGTCGTCACCTTCATGGAAGAAATATGGAAGTG-3′
Chi18A-NPr5′	5′-TTCCAAGGTCCACTGGGATCCACTAGTCAGGACGACTCTCTTCCGACAAAAAG-3′
Chi18A-CF3′	5′-GGGACGTCGACGCGTTTACTTGTCATCGTCATCCTTGTAGTCGTCACCATCATCAACAGGTATATTGTC-3′
Cel5,26H-NPr5′	5′-TTCCAAGGTCCACTGGGATCCACTAGTAATTACAACAGTGGTTTAAAAATCG-3′
Cel5,26H-CF3′	5′-GGGACGTCGACGCGTTTACTTGTCATCGTCATCCTTGTAGTCGTCACCTATGGGTATTTCACTGATGGC-3′

Cel124A-NPr5′	5′-TTCCAAGGTCCACTGGGATCCACTAGTTGGAATAAGGCAGTTATTGGAGATG-3′
Cel124A-CF3′	5′-ACGTCGACGCGTTTACTTGTCATCGTCATCCTTGTAGTCGTCACCGTAATAAATCTCCCATGGATTGC-3′
GH9 (Cthe_2761)-NPr5′	5′-TTCCAAGGTCCACTGGGATCCACTAGTGAAGAGCCAAAATTTAACTATGTAG-3′
GH9 (Cthe_2761)-CF3′	5′-ACGTCGACGCGTTTACTTGTCATCGTCATCCTTGTAGTCGTCACCCTGCTCTACGGGGAACTTATCTAT-3′
GH9 (Cthe_0433)-NPr5′	5′-TTCCAAGGTCCACTGGGATCCACTAGTGCTAATGTGGAATACAACTATGC-3′
GH9 (Cthe_0433)-CF3′	5′-ACGTCGACGCGTTTACTTGTCATCGTCATCCTTGTAGTCGTCACCTTTATCGGAATAACCTCGATTG-3′
Man26B-NPr5′	5′-TTCCAAGGTCCACTGGGATCCACTAGTGCATATTCCCTTCCTGTGGACG-3′
Man26B-CF3′	5′-ACGTCGACGCGTTTACTTGTCATCGTCATCCTTGTAGTCGTCACCCTTTTGTGTAACGGGAAATTTTGT-3′
GH53 (Cthe_1400)-NPr5′	5′-TTCCAAGGTCCACTGGGATCCACTAGTGCTCCAACTTTTGCAAAGGGGGCTG-3′
GH53 (Cthe_1400)-CF3′	5′-ACGTCGACGCGTTTACTTGTCATCGTCATCCTTGTAGTCGTCACCTTTTACAGGAAGTGATGGTATGG-3′
Gal43A-NPr5′	5′-TTCCAAGGTCCACTGGGATCCACTAGTGCAGAAGGGGTTATAGTCAAC-3′
Gal43A-CF3′	5′-ACGTCGACGCGTTTACTTGTCATCGTCATCCTTGTAGTCGTCACCCAAATCCACTTCCATAAGCAAG-3′
Rgl11A-NPr5′	5′-TTCCAAGGTCCACTGGGATCCACTAGTTCAACCAAGTATGGGGATCTC-3′
Rgl11A-CF3′	5′-ACGTCGACGCGTTTACTTGTCATCGTCATCCTTGTAGTCGTCACCCGGCACAAGGTAAATATTTGGCTT-3′
Rgae12A-NPr5′	5′-TTCCAAGGTCCACTGGGATCCACTAGTGCCCCGAATGAATACAAGTTTG-3′
Rgae12A-CF3′	5′-ACGTCGACGCGTTTACTTGTCATCGTCATCCTTGTAGTCGTCACCATTGGTTTTCAAATACTTCGC-3′
Xyn5A-NPr5′	5′-TTCCAAGGTCCACTGGGATCCACTAGTAGCCCGCAACGTGGCCGGCCGCGT-3′
Xyn5A-CF3′	5′-ACGTCGACGCGTTTACTTGTCATCGTCATCCTTGTAGTCGTCACCTAAAGTAGTTATGACACGGAG-3′
Xyn30A-NPr5′	5′-TTCCAAGGTCCACTGGGATCCACTAGTGCAGAAACGGCAACAATCAACTTG-3′
Xyn30A-CF3′	5′-ACGTCGACGCGTTTACTTGTCATCGTCATCCTTGTAGTCGTCACCTGATTCTACAGGAAATTTATC-3′
